# Effect of delayed cord clamping and cord milking on cerebral oxygenation and cardiovascular function: a secondary analysis of the PCI trial

**DOI:** 10.1007/s00431-026-06783-z

**Published:** 2026-02-16

**Authors:** Simone Pratesi, Stefano Ghirardello, Cristiana Germini, Miria Natile, Stefania Vedovato, Giovanna Mescoli, Roberta Corbetta, Flavia Petrillo, Anna Lavizzari, Silvia Perugi, Luca Boni, Carlo Dani

**Affiliations:** 1 Department of Neuroscience, Psychology, Drug Research and Child Health, University of Florence, Florence, Italy; 2 Division of Neonatology, Careggi University Hospital, Florence, Italy; 3 SC Terapia Intensiva Neonatale E Neonatologia Fondazione IRCCS Policlinico San Matteo, Pavia, Italy; 4 Department of Pediatrics, Santa Maria Della Misericordia Hospital, Perugia, Italy; 5 Neonatal Intensive Care Unit, Division of Neonatology, “Infermi” Hospital, Rimini, Italy; 6 Department of Pediatrics, Neonatal Intensive Care Unit, San Bortolo Hospital, Vicenza, Italy; 7 Neonatal Intensive Care Unit, Department of Womens and Childrens Health, Maggiore Hospital, Bologna, Italy; 8 Neonatal Intensive Care Unit, Fondazione IRCCS San Gerardo Dei Tintori, Monza, Italy; 9 Neonatal Intensive Care Unit, Department of Womens and Childrens Health, Di Venere’ Hospital, Bari, Italy; 10 Department of Mother and Infant Science, Fondazione IRCCS Ca Granda Ospedale Maggiore Policlinico, University of Milan, Milan, Italy; 11 IRCCS Ospedale Policlinico San Martino, Genoa, Italy

**Keywords:** Delayed cord clamping, Cord milking, Cerebral oxygenation, Cardiac function, Preterm infant

## Abstract

Umbilical cord clamping management may affect cerebral oxygenation (rSO_2_C), but previous studies have only investigated effects in the first minutes of life. Our objective was to determine whether delayed cord clamping (DCC) and umbilical cord milking (UCM) differently affect cerebral oxygenation and cardiovascular function in the first 24 h of life in preterm infants. A post-hoc secondary outcome analysis of a multicentre prospective randomised clinical trial (PCI), conducted between April 2016 and February 2023 at 8 Italian neonatal intensive care units. The present ancillary study included preterm infants with 23^+0^–29^+6^ 30 weeks’ gestation. One hundred and five infants received DCC and 104 UCM during resuscitation. Cerebral regional tissue oxygenation (rSO_2_C) was measured by near-infrared spectroscopy (NIRS) at 3 (T_3h_), 6 (T_6h_), 12 (T_12h_), 18 (T_18h_), and 24 (T_24h_) hours of life. Cardiovascular function was assessed by echocardiography within the first 24 h of life. We found that rSO_2_C was higher at T_3h_ [79 (76–84) vs. 78% (74–82), *P* = 0.04)] and T_12h_ [79 (76–83) vs. 78% (74–80), *P* = 0.01] in the DCC than in the UCM group. Left ventricular output (LVO) was lower [196 (182–301) vs. 232 (182–301) ml/Kg/min, *P* = 0.02] in the DCC than in the UCM group, while right ventricular output (RVO) and superior vena cava (SVC) flow were similar. The need for dopamine was higher (26 vs. 23%, *P* = 0.02) in the in the DCC than in the UCM group.

*Conclusion*: rSO_2_C was transiently higher in preterm infants resuscitated with DCC in comparison with UCM, but this difference was not clinically relevant. The lower LVO value in the DCC group compared to the UCM group deserves further studies to be confirmed and interpreted.

**Supplementary Information:**

The online version contains supplementary material available at 10.1007/s00431-026-06783-z.

**Table:** 

**What is Known:** • *Umbilical cord clamping management may affect cerebral oxygenation (rSO*_*2*_*C)*. • *Previous studies showed no differences of brain oxygenation in the first minutes of life due to different timing of cord clamping or umbilical cord milking (UCM)*.
**What is New:** • *We showed that rSO*_*2*_*C was transiently higher in preterm infants resuscitated with delayed cord clamping (DCC) in comparison with UCM during the first day of life*. • *We found a lower left ventricular output in the DCC group compared to the UCM group, which deserves confirmation and interpretation*.

## Introduction

It has been demonstrated that delayed cord clamping (DCC) of 30–60 s improves outcomes in preterm infants not requiring resuscitation in the delivery room compared with immediate cord clamping (ICC) [1–3]. However, ICC after birth continues to be performed, mainly in case of cesarean section, to allow a quick resuscitation [4–7]. When DCC is not feasible, umbilical cord milking (UCM) is considered an alternative procedure to be performed at birth, because it is associated with better outcome in preterm infants compared with ICC [3, 8–12].

A recent meta-analysis concluded that DCC may result in little to no difference in the outcome of severe intraventricular hemorrhage (IVH) compared to UCM [13], but some concerns remain about an increased risk of IVH in infants born < 28 weeks of gestation resuscitated with UCM [14]. Therefore, monitoring of cerebral oxygenation by near-infrared spectroscopy (NIRS) during immediate transition has become increasingly widespread [15]. Cerebral regional tissue oxygenation (rSO_2_C) during the crucial postnatal period depends on several factors, including hemodynamic, metabolic, respiratory, and perinatal factors; addressing these with targeted interventions can potentially help prevent brain injury [16].

Perinatal factors which can affect rSO_2_C also include the management of cord clamping and some studies investigated the effects of ICC, DCC, and UCM on brain oxygenation in very [17, 18] and extremely [19] preterm infants. These studies [17–19] did not show significant differences of brain oxygenation related to different timing of cord clamping or UCM. However, they differ in their design, size and gestational age of the population, and length of the study period [17–19] and, therefore, their results cannot be considered conclusive. In particular, the duration of rSO_2_C monitoring is often limited to the first minutes of life, and the possible subsequent effects have been little studied.

We recently published a randomized controlled study which compared the outcome of preterm infants born at less than 30 weeks’ gestation resuscitated at birth with a 180-s DCC with that of patients resuscitated with UCM [20]. We found that the survival without grade 3 to 4 intraventricular hemorrhage (IVH) and bronchopulmonary dysplasia (BPD) was not significantly different between the groups [20]. In this study we also evaluated the cerebral oxygenation and cardiovascular functions of studied infants [20].

### Aim of the study

The aim of this present ancillary analysis was to evaluate possible differences in rSO_2_C and cardiovascular function during the first day of life in preterm infants who were resuscitated with DCC or UCM.

## Material and methods

### Study design

This is a post-hoc secondary outcome analysis of a multicentre prospective randomised clinical trial (PCI), conducted between April 2016 and February 2023 at 8 Italian neonatal intensive care units. The aim of the study was to evaluate differences in rSO_2_C within the first day of life resuscitated with DCC or UCM. The study was approved by local ethics committees and written parental consent was obtained before birth. The prospective randomised-controlled trial was registered at ClinicalTrials.gov (NCT02671305). The results of the primary outcome of the PCI trial have already been published elsewhere [20].

### Study population

Eligible newborns were those with a gestational age between 23^+0^ and 29^+6^ weeks, whose parents signed the informed consent form. Exclusion criteria were twin or multiple births, placental and cord abnormalities, major congenital malformations, hydrops fetalis, and maternal severe compromise at delivery.

Delivery room interventions were performed as described in the main study [20]. In the UCM group, 20 cm of the intact cord was squeezed over 2 s, repeated for a total of 4 times, and then the cord was clamped and cut within 20 s of life, whereas in the PCI group the cord was clamped at 180 s without milking [20].

### Cerebral oxygenation and cardiovascular function assessment

rSO_2_C was measured by NIRS (SenSmart™ X-100, Nonin Medical Inc, Plymouth, MN, USA) at 3 (T_3h_), 6 (T_6h_), 12 (T_12h_), 18 (T_18h_), and 24 (T_24h_) hours of life.

To assess cardiovascular function, right ventricular output (RVO) and left ventricular output (LVO), pulmonary arterial pressure (PAP), superior vena cava (SVC) flow, and left-to-right ductal shunting were measured by echocardiography within the first 24 h of life. Immediately thereafter, pulsatility and resistance indices in the anterior cerebral artery were measured by cerebral ultrasound. Mean systemic arterial pressure (SAP) was recorded at T_3h_, T_6h_, T_12h_, T_18h_, T_24h_, as well as the need for inotropes (i.e.: dopamine, dobutamine). Echocardiographic measurements were performed on all infants within the first 24 h of life of age by clinicians who were blinded to infant’s randomization and were not involved in patient care.

### Data collection

Main clinical characteristics and occurrence of prematurity complications were reported. Patent ductus arteriosus (PDA) was reported when treatment was required due to echocardiographic findings of hemodynamic significance [21]. The diagnosis of BPD was based on the definition of moderate and severe BPD by Jobe et al. [22], IVH was diagnosed with the criteria of Papile et al. [23], necrotizing enterocolitis (NEC) was diagnosed with the criteria of Bell et al. [24], and periventricular leukomalacia (PVL) was diagnosed according to the criteria of de Vries et al. [25].

### Endpoint of the study

The primary endpoint was the evaluation of changes in rSO_2_C during the first 24 h of life in infants resuscitated with DCC compared to UCM. The secondary endpoint was the comparison of cardiovascular function measured in the first 24 h of life in infants resuscitated with DCC or UCM.

### Statistical analysis

This was a post-hoc exploratory analysis of data obtained during the PCI trial [20], therefore, no power calculation was conducted.

Patients’ clinical characteristics were described as mean ± SD, rate and percentage, or median and interquartile range (IQR). For rSO_2_C we calculated the median and IQR from selected 5-min periods which were chosen at the end of T_3h_, T_6h_, T_12h_, T_18h_, T_24h_ [26]. We made this choice to obtain the highest stability of NIRS signal. However, sometimes this was not possible due to the occurrence of unwanted artifacts (generally infant movements): in this case the 5-min period without artifacts closest to the end of the study period was selected.

Univariable statistical analysis was performed using the Wilcoxon rank-sum test for continuous variables and the χ^2^ test or Fisher exact test when appropriate for categorical variables. A 2-sided P value < 0.05 was considered statistically significant. Serial measurements of studied variables were compared by repeated-measures analysis of variance (ANOVA).

## Results

All infants included in the PCI trial [20] were also included in the final ancillary analysis. One hundred and five infants received DCC and 104 UCM during resuscitation. Demographic characteristics were similar between groups (Table 1).

**Table 1:** Neonatal demographics by treatment group. Mean ± SD, rate and (%), or median and (IQR)

	DCC (*n* = 105)	UCM (*n* = 104)
Gestational age (wks)	26.7 ± 1.7	26.6 ± 1.7
23^+0^–26^+6^ wks	43 (41)	45 (43)
Birth weight (g)	942 ± 246	898 ± 270
Female	45 (43)	45 (43)
Antenatal steroids	102 (97)	100 (96)
Cesarean delivery	54 (51)	64 (61)
Apgar score at 5 min	8 (7–9)	8 (7–9)
Peak FiO_2_ in the delivery room	50 (40–100)	50 (30–100)
Peak hemoglobin in first 24 h of life (g/dL)	18.3 (16–20)	17.6 (16–20)
Peak hematocrit in first 24 h of life (%)	51 (46–60)	51 (46–58)
Noninvasive ventilation	99 (94)	93 (89)
Mechanical ventilation	60 (57)	49 (47)
Patent ductus arteriosus	53 (50)	51 (49)
Early onset Sepsis	3 (3)	8 (8)
Late onset Sepsis	30 (28)	36 (35)
Bronchopulmonary dysplasia	49 (47)	51 (49)
Intraventricular hemorrhage	26 (25)	26 (25)
3–4 grade hemorrhage	8 (8)	12 (12)
Necrotizing enterocolitis	2 (2)	2 (2)
Periventricular leukomalacia	2 (2)	4 (4)
Retinopathy of prematurity	21 (20)	28 (27)
Mortality	9 (9)	13 (13)
Intrauterine growth restriction	18 (17)	22 (21)
Preterm premature rupture of membranes	33 (31)	39 (37)
Maternal chorioamnionitis	19 (18)	14 (13)
Preeclampsia	18 (17)	22 (21)

**Fig. 1:**
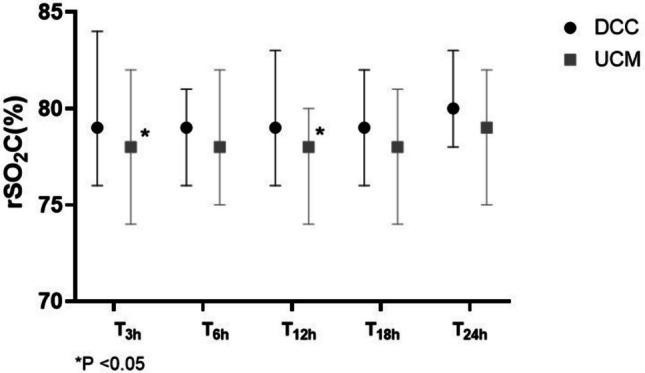
Changes in cerebral regional tissue oxygenation (rSO_2_C) measured by near-infrared spectroscopy (NIRS) at 3 (T_3h_), 6 (T_6h_), 12 (T_12h_), 18 (T_18h_), and 24 (T_24h_) hours of life in in neonates resuscitated with delayed umbilical cord clamping (black circle, DCC) or umbilical cord milking (gray square, UCM)

We found that rSO_2_C was higher at T_3h_ [79 (76–84) vs. 78% (74–82), *P* = 0.04)] and T_12h_ [79 (76–83) vs. 78% (74–80), *P* = 0.01] in the DCC than in the UCM group. There was a trend toward the increase of rSO_2_C during the first 24 h of life in both the DCC and UCM groups, but it was not statistically significant. Similarly, the PI and the RI were similar between the groups, as well as the occurrence of absent diastolic flow in the anterior cerebral artery (Table 2, Fig. 1, Fig. S1).

**Table 2:** Changes of cerebral regional tissue oxygenation (rSO_2_C) at the different data points of the study, pulsatility and resistance indices and absence of diastolic flow in anterior cerebral artery (ACA) in infants resuscitated with delayed cord clamping (DCC) or umbilical cord milking (UCM). Median and (IQR) and rate

	DCC (*n* = 105)	UCM (*n* = 104)	*P*
rSO_2_C-T_3h_	79 (76–84)	78 (74–82)	0.04
rSO_2_C-T_6h_	79 (76–81)	78 (75–82)	0.68
rSO_2_C-T_12h_	79 (76–83)	78 (74–80)	0.01
rSO_2_C-T_18h_	79 (76–82)	78 (74–81)	0.10
rSO_2_C-T_24h_	80 (78–83)	79 (75–82)	0.12
P	0.500	0.500	
Resistance index in ACA	1.6 (1.3–2.4)	1.8 (1.3–2.5)	0.72
Pulsatility index in ACA	0.72 (0.65–0.80)	0.78 (0.65–0.86)	0.11
Absent diastolic flow in ACA	13 (12)	18 (17)	0.58

Echocardiographies were performed at 10.4 ± 7.9 and 12.9 ± 7.7 h of life in the DCC and UCM groups, respectively. LVO was lower [196 (182–301) vs. 232 (182–301) ml/Kg/min, *P* = 0.02] in the DCC than in the UCM group, while RVO and SVC flow were similar. Of note, the PAP did not differ between the groups, which is consistent with a similar occurrence of left-to-right ductal shunt in this group. SAP values were similar between the groups and did not vary during the study period (Fig. S1). However, the need for dopamine was higher (26 vs. 23%, *P* = 0.02) in the DCC than in the UCM group (Table 3).

**Table 3:** Cardiovascular function parameters and need for inotropes in infants resuscitated with delayed cord clamping (DCC) or umbilical cord milking (UCM). Median and (IQR), rate and (%)

	DCC (*n* = 105)	UCM (*n* = 104)	*P*
Superior vena cava flow (ml/Kg/min)	91 (64–139)	94 (68–139)	0.74
Pulmonary arterial pressure (mmHg)	25 (18–40)	28 (20–45)	0.36
Right ventricular output (ml/Kg/min)	262 (206–354)	291 (210–408)	0.36
Left ventricular output (ml/Kg/min)	196 (182–301)	232 (182–301)	0.02
Left-to-right ductal shunt	86 (82)	79 (76)	0.14
Systemic mean arterial pressure (mmHg):			
T_3h_	35 (30–41)	35 (30–41)	0.89
T_6h_	36 (32–43)	37 (31–43)	0.69
T_12h_	37 (32–42)	38 (32–45)	0.36
T_18h_	39 (34–43)	38 (34–46)	0.91
T_24h_	40 (35–47)	40 (35–46)	0.99
Dopamine in the first 24 h	27 (26)	14 (13)	0.02
Dobutamine in the first 24 h	8 (8)	6 (6)	0.59

## Discussion

This is a post-hoc secondary outcome analysis of a multicentre prospective randomized clinical trial in which rSO_2_C and cardiovascular function during the first 24 h of life were compared in preterm infants born at < 30 weeks of gestation who were resuscitated with DCC or UCM. We found that rSO_2_C was higher at T_3h_ and T_12h_ in the DCC than in the UCM group. Moreover, LVO was lower and the need for inotropes was higher in the DCC than in the UCM group.

Previous studies evaluated the effect of umbilical cord management on cerebral oxygenation [17–19]. Perme et al. studied 572 preterm infants with a gestational age < 32 weeks and found that a DCC > 60 s did not affect rSO_2_C during the first 15 min of life compared to an ICC < 30-s and a DCC 30–60 s [18]. Katheria et al. studied 56 preterm infants born between 23 and 27 weeks of gestation and found similar rSO_2_C in the first 10 min of life in infants resuscitated with a DCC > 60 s or UCM [19]. Both studies [18, 19] assessed rSO_2_C earlier and after a shorter DCC than ours, and therefore their findings are complementary to our results. Finn et al. studied 45 preterm infants with a gestational age < 32 weeks and found that ICC, DCC > 60-s, and UCM were associated with similar rSO_2_C levels at 12 and 24 h of life [17]. These results disagree our findings and this may depend by several causes, such as the small size of Finn’s population and the different used device [17]. However, the increase of rSO_2_C at 3 and 12 h of life that we observed, although statistically significant, was not clinically relevant since it was transient, and the measured values fell within the normal range of cerebral oxygenation [27]. Moreover, these findings are reassuring with respect to a possible large increase in cerebral blood flow and, therefore, in the risk of IVH in neonates assisted with UCM [20]. Consistently, the similar values of PI and RI and the similar incidence of absent diastolic flow in the ACA, as well as the similar frequency of IVH [20] suggest that DCC and UCM did not differently affect cerebral perfusion in our population. It is difficult to explain why infants treated with DCC showed this increase in rSO_2_C, since variables that could have explained it, such as hemoglobin/hematocrit values and SVC flow, were similar between the groups. On the other hand, there may have been a component of cerebral autoregulation that may have affected our results. Interestingly, infants in the DCC group were treated more frequently with dopamine, which could suggest a beneficial effect of this inotrope on cerebral perfusion. However, this remains a speculation because the effect of dopamine on cerebral autoregulation and blood flow is not fully understood [28].

We found that in the first 24 h of life LVO was lower in the DCC than in the UCM group, while RVO and SVC flow did not vary. A recent meta-analysis partially agreed with these results and reported a trivial reduction in LVO in infants resuscitated with DCC vs. UCM, a range of large reduction to a trivial reduction in RVO, and a range of small reduction to moderate increase in SVC flow between the two interventions with an evidence of very low quality [13]. On the other hand, 3 RCTs with only 208 participants were meta-analyzed [13]. The increase of LVO in the UCM group may depend on larger placental blood transfusion which increases cardiac preload and stroke volume and improves systemic perfusion, as it may be suggested by the lower need for inotropes. However, LVO in the presence of a ductal shunt may measure both systemic blood flow and the flow across the patent ductus arteriosus (PDA) and, therefore, may be significantly overestimated [29]. Conversely, SVC flow represents cardiac input and, therefore, is an accurate measure of systemic blood flow because is not affected by the presence of fetal shunts [29]. Thus, although our results seem reassuring regarding the safety of the UCM, further studies are necessary to confirm and interpret our findings on the effects of cord management on cardiovascular function.

There are some limitations to this study. We did not calculate oxygen extraction fraction because SpO_2_ was not recorded simultaneously with rSO_2_C. However, differences in cerebral oxygenation between groups were minimal, and this parameter was not necessary to interpret large variations. We did not measure rSO_2_C in the first minutes of life and, the echocardiographic evaluation of cardiovascular function was performed only once in the first 24 h. Furthermore, the exact timing of IVH development was not recorded. However, previous studies did not report differences in rSO_2_C in the first minutes of life between DCC and UCM [17–19], and scheduling additional echocardiograms was considered too complicated in a multicenter study [20]. However, studying rSO_2_C and cardiovascular function during the first 24 h of life remains very important due to the paucity of data in the literature and especially because 25 and 50% of IVH develop in the first and second day of life, respectively [30].

In conclusion, we found that rSO_2_C was transiently higher during the first 24 h after birth in preterm infants resuscitated with DCC in comparison with UCM, but this difference was not clinically relevant. We found that LVO was lower on the first day of life in the DCC than in the UCM group, while SVC flow was similar. Although our results contribute to the understanding of the effect of different umbilical cord management on cerebral oxygenation and cardiovascular function during the first hours of life, further research is needed to confirm and complete our findings.

## Supplementary Information

Below is the link to the electronic supplementary material.

Supplementary file1 (JPG 49 KB)

## Data Availability

Data are available on reasonable request.

## Abbreviations

DCC: Delayed cord clamping
BPD: Bronchopulmonary dysplasia
ICC: Immediate cord clamping
IVH: Intraventricular hemorrhage
LVO: Left ventricular output
NEC: Necrotizing enterocolitis
NIRS: Near-infrared spectroscopy
PDA: Patent ductus arteriosus
PAP: Pulmonary artery pressure
PVL: Periventricular leukomalacia
RDS: Respiratory distress syndrome
rSO_2_C: Cerebral regional tissue oxygenation
RVO: Right ventricular output
SAP: Systemic arterial pressure
SVC: Superior vena cava
UCM: Umbilical cord milking
