# Supplementary figures and images for: Effect of delayed cord clamping and cord milking on cerebral oxygenation and cardiovascular function: a secondary analysis of the PCI trial
Source: Eur J Pediatr. 2026 Feb 16;185(3):133. doi: 10.1007/s00431-026-06783-z (PMC12909620; doi:10.1007/s00431-026-06783-z)

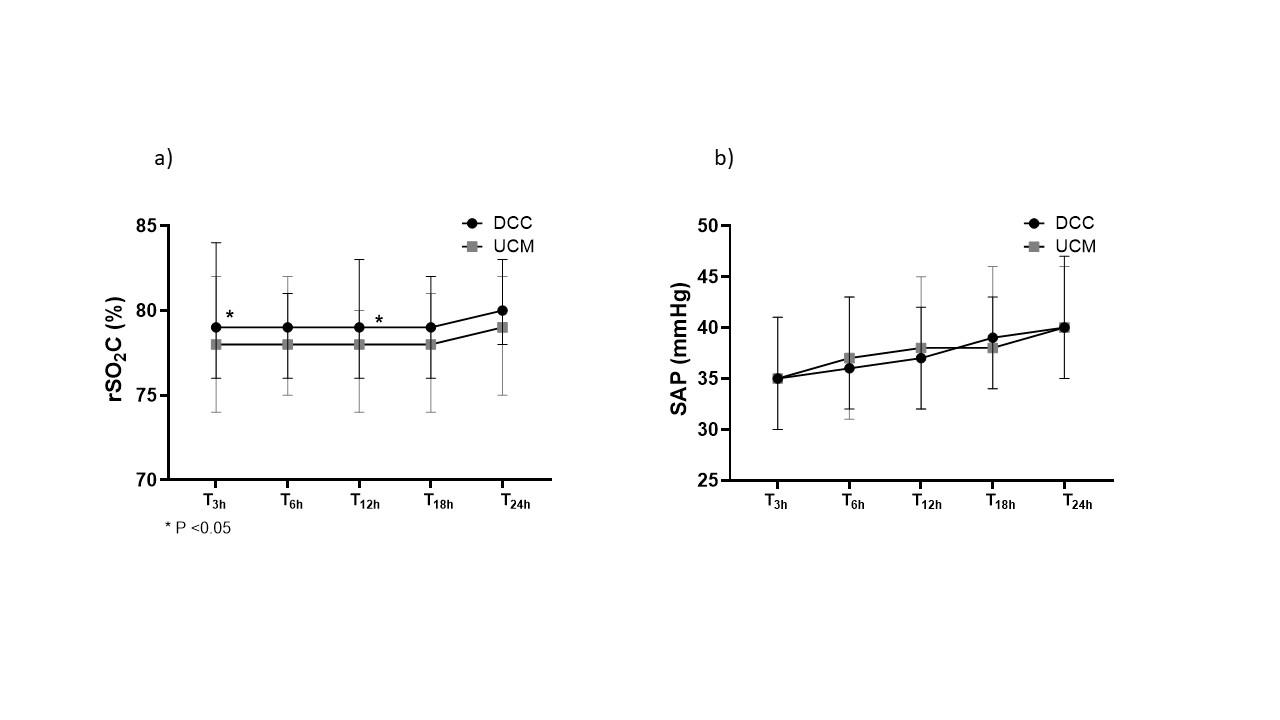

Supplement: Supplementary file 1 — Supplementary file1 (JPG 49 KB) [file 431_2026_6783_MOESM1_ESM.jpg]
